# Case Series: Combination of dupilumab and omalizumab as a way to reduce dupilumab-associated adverse events in severe atopic dermatitis

**DOI:** 10.3389/falgy.2025.1696897

**Published:** 2026-01-16

**Authors:** Daria S. Fomina, Olga A. Mukhina, Elizaveta L. Sedova, Marina S. Lebedkina, Elena N. Bobrikova, Alexander V. Karaulov, Mariana A. Lysenko, Harald Renz

**Affiliations:** 1 State Budgetary Healthcare Institution of Moscow City “Moscow Clinical Research Center 7 Hospital No. 52 of the Moscow City Health Department”, Moscow, Russia; 2 Federal State Autonomous Educational Institution of Higher Education I.M.Sechenov First 12 Moscow State Medical University of the Ministry of Health of the Russian Federation 13, Sechenovskiy University, Moscow, Russia; 3 Research Institute of Healthcare Organization and Medical Management of Moscow Department 10 of Healthcare, Moscow, Russia; 4 Pirogov Russian National Research Medical University, Moscow, Russia; 5 Philipps University of Marburg, Marburg, Germany

**Keywords:** dupilumab, atopic dermatitis (AtD), conjunctivitis, face and neck dermatitis, dupilumab-associated adverse effects, anti-IgE therapy, omalizumab

## Abstract

Dupilumab is a fully human monoclonal antibody directed against the interleukin-4 receptor subunit α, the common chain of the IL-4 and IL-13 receptors. Dupilumab has been demonstrated to be highly effective in the treatment of severe atopic dermatitis, asthma, and chronic rhinosinusitis with nasal polyps. Despite the favorable tolerability of dupilumab, several publications and reviews have reported various adverse events, including conjunctivitis, keratitis, and face and neck treatment-resistant dermatitis, which reduce patients’ quality of life and may necessitate the cancellation of therapy. The current standard of care for severe chronic diseases involves a collaborative approach between the patient and their physician. The majority of patients who respond well to this type of systemic atopic dermatitis (AtD) therapy are reluctant to interrupt dupilumab treatment due to the previously mentioned adverse events. The combination of dupilumab and anti-IgE therapy (omalizumab) may be beneficial for individuals who undergo dupilumab treatment for AtD and experience severe adverse events.

## Introduction

Dupilumab is a monoclonal antibody that blocks the signaling of key T helper 2 (Th2)-inflammatory cytokines [interleukin-4 (IL-4) and IL-13] by specifically binding to IL-4Ra, a common chain of the IL-4 and IL-13 receptor complexes (1). Numerous studies have confirmed the efficacy of dupilumab in the treatment of severe atopic dermatitis (AtD), asthma, and chronic rhinosinusitis with nasal polyps (2–6).

Despite the favorable tolerability profile, a number of publications have highlighted a range of adverse events (AEs) associated with dupilumab (7). The most commonly reported AEs in AtD were eye lesions (namely, conjunctivitis), keratitis, blepharitis, itchy eyes, and dry eyes (8). The pathogenesis of these adverse events remains controversial, but is most likely multifactorial (9). The majority of randomized placebo-controlled studies have demonstrated a higher incidence of conjunctivitis in patients with AtD treated with dupilumab (8.6%–22.1%) compared to the placebo (2.1%–11.1%). In asthma, randomized placebo-controlled clinical trials of dupilumab only showed low rates of conjunctivitis (0%–1.7%), comparable with that of placebo (0%–3.3%) (10). In patients with chronic rhinosinusitis with nasal polyps treated with dupilumab, no AEs related to the eyes have been recorded (11).

The incidence of eye diseases associated with dupilumab was found to be higher during the first 4 months of the treatment, with a subsequent decline in new cases over time (12). In a 3-year open clinical trial, 1.4% (3/217) of patients discontinued dupilumab due to conjunctivitis (13). Similar outcomes were also observed in a 52-week BioDay study, in which 2.4% (5/210) of patients discontinued dupilumab due to the development of conjunctivitis (14).

Recent publications caution against the discontinuation of dupilumab, despite the AEs, including those in the eyes (15, 16). This underscores the need for the development of effective management strategies for such patients in routine clinical practice.

Erythema of the face and neck has recently been reported as an AE during dupilumab treatment in several cases in routine clinical practice (17–19), although it was not reported during Phase III clinical trials (6). A systematic review of 16 studies of dupilumab-associated erythema of the face and neck by Jo et al. identified 101 patients who reported this AE. The proposed etiology of face and neck erythema includes rosacea, allergic contact dermatitis, and head and neck dermatitis. Eleven out of the 101 patients (11%) discontinued dupilumab due to these AEs. However, thanks to the modern concept of shared decision-making, most of the patients continued the therapy despite the development of AEs (20).

Despite the lack of reliable data on the etiology of dupilumab-associated face and neck dermatitis, some authors have highlighted the potential role of an increase in serum IgE specific to *Malassezia* prior to dupilumab treatment. In a study by Kozera et al., a significant increase in IgE specific to *Malassezia* was observed in patients with dupilumab-associated face and neck dermatitis compared to those without this AE (median 31.95 kU/L vs. 2.27; *P* = 0.005) (21). The level of *Malassezia*-specific IgE may serve as a potential biomarker of the safety of atopic dermatitis treatment with dupilumab (21, 22) and may be a new therapeutic target for the treatment of dupilumab-associated AEs. The use of monoclonal antibody combinations could provide new opportunities for patients and expand the indications for this type of biological therapy. However, the safety of combination therapy is currently poorly understood (23, 24). The effectiveness of combined biological therapy also needs further research.

The following clinical case series illustrates the efficacy of anti-IgE therapy in patients who experienced adverse events while undergoing dupilumab treatment for severe atopic dermatitis (Supplementary Table S1).

## Clinical case 1

Patient I (male, 40 years old) presented to an allergist-immunologist in March 2023 with complaints of skin rashes accompanied by severe itching, periodic shortness of breath, rhinitis, and conjunctivitis following contact with causally significant allergens.

Since early childhood, the patient has exhibited the development of skin rashes accompanied by itching. Dermatologists diagnosed the atopic dermatitis, and the administration of topical glucocorticosteroids (GCSs) was prescribed with an efficacious outcome. At the age of 35, a stressful situation provoked a pronounced exacerbation of generalized rashes accompanied by painful itching. In order to provide relief, systemic glucocorticosteroids (SGCs), phototherapy, and plasmapheresis were prescribed, with a short-term positive effect.

The patient has experienced rhinoconjunctivitis during the pollination season and when in contact with animal hair since childhood. The dermatitis was exacerbated by the consumption of certain types of meat, nuts, and chicken eggs. Furthermore, the patient has experienced swelling in the oral cavity when consuming fish and seafood. Despite this, the patient did not seek the advice of an allergist and instead self-treated the symptoms with antihistamines.

At the age of 7, the patient first exhibited symptoms of respiratory distress, including shortness of breath, wheezing, whistling in the chest, and paroxysmal coughing, particularly during periods of physical exertion and during the pollination season. The pulmonologist diagnosed the patient with asthma and prescribed various inhaled GCSs as background therapy. His current treatment regimen includes a budesonide/formoterol 160/4.5 inhaler, with two doses administered twice a day, and tiotropium bromide 5 mcg taken daily. Short-acting β2-agonists (SABA) are used 5–7 times per week.

The molecular diagnosis of the allergic component was conducted using the ALEX^2^® Allergy Test, which revealed sensitization to a variety of allergens, including household (house dust mites and barn mites), epidermal (dog, cat, mouse, rabbit, rat, and horse), fungal (*Malassezia* and *Alternaria*), pollen (pollen of trees, cereals and weeds), and numerous food allergens (including fish and seafood) (Supplementary Table S1; Supplementary Figure S1.1).

On dupilumab initiation, the skin process was assessed according to SCORing Atopic Dermatitis index, Eczema Area and Severity Index, body surface area, Patient Oriented Eczema Measure, and Dermatology Life Quality Index (Supplementary Table S2). Asthma was assessed using the Asthma Control Test (ACT), 16 points; Asthma Control Questionnaire (ACQ5), 4 points; and Asthma Quality of Life Questionnaire (AQLQ), 97 points, which corresponds to uncontrolled asthma with a significantly lowered quality of life (QoL).

Dupilumab treatment was initiated, with the patient initially receiving a single injection of 600 mg of dupilumab subcutaneously (s.c.), followed by 300 mg s.c. once every 2 weeks. The patient’s total IgE level was 2,786 IU/mL at the initiation of therapy. Despite this high level of total IgE, the level of IgE specific to *Malassezia* (Mala s 6) of 2.61 kUA/L was considered clinically significant, taking into account the reference value of 1.0–5.0 kUA/L, which is characteristic of a moderate level according to the ALEX^2^ allergen component test. Within 6 months of starting therapy, a positive response was observed, with gradual regression of the rashes on the trunk and extremities, decreased itching, and an absence of asthma attacks. There was no need for SABA or SGC therapy. Skin dryness remained. However, 4 months after the initiation of dupilumab, conjunctivitis developed and persisted despite the administration of an ophthalmologist-prescribed therapy (topical antihistamines and topical GCS) (Supplementary Figure S1A).

After 6 months of dupilumab treatment, the medical commission added 300 mg of omalizumab s.c. per month. The dose of the medication was chosen independently of the total IgE level and the patient's body weight, based on the limited data of positive results in other studies (25–29) (Supplementary Table S1). Anti-IgE therapy was found to be effective in resolving the conjunctivitis after 2 months of treatment with omalizumab (Supplementary Figure S1B, scale for clinical case 1).

## Clinical case 2

Patient T (male, 23 years old) presented with predominant complaints of skin rashes accompanied by painful itching, episodic asthma attacks, and rhinoconjunctivitis upon contact with allergens.

Patient has a history of atopic dermatitis since early childhood, with no remission despite ongoing care by an allergist and dermatologist.

Since the beginning of 2020, the patient has observed progressive deterioration, with the rashes spreading over his skin, accompanied by painful skin itching. Topical GCS (betamethasone), calcineurin inhibitors in the facial area (pimecrolimus), and emollients were employed with a short-term effect. Prior to the administration of monoclonal antibodies, a 14-day course of an SGC (dexamethasone 16 mg/day) was initiated.

The patient has been diagnosed with allergic rhinoconjunctivitis since childhood. Contact with household dust, animal hair, pollen, seasonal allergens (in April–September), and damp and humid rooms were identified as the triggers. Oral itching and exacerbation of dermatitis have been observed when eating potatoes, tomatoes, bananas, nuts, and stone fruits. Fish has been found to cause bronchospasm.

Since 2002, the patient has been diagnosed with asthma. The background therapy included salmeterol/fluticasone 25/250 mcg administered via inhalation twice daily, with the need for SABA use up to 1–2 times a day.

A blood test indicated an elevated eosinophil count (up to 580 cells/mL) and a total IgE level over 2,000 IU/mL, which exceeded the titrable range. Additionally, his forced expiratory volume in 1 s was 81% (4.14 L).

Molecular allergocomponent diagnostics using the ALEX^2^ allergochip demonstrated sensitization to a variety of allergens, including household (house dust mites and barn mites), epidermal (dog, cat, hamster, and horse), fungal (*Malassezia*, *Aspergillus fumigatus*, and *Alternaria alternata*), pollen (tree and weed pollen), food (seafood, nuts, apple, and strawberry), and hymenopteran venom (ant and cockroach) allergens (Supplementary Table 1; Supplementary Figure S1.2).

Upon dupilumab initiation, the AtD course and asthma were assessed comprehensively (Supplementary Table S1); the asthma was very poorly controlled according to the ACT, 8 points, and ACQ5, 2.8 points; and his quality of life was low, according to AQLQ, 87 points.

Dupilumab therapy was started with a 600 mg subcutaneous loading dose, after which the patient received 300 mg subcutaneous injections at 2-week intervals. After 6 months of therapy, a positive response was observed, including gradual rash regression, the absence of asthma attacks, and no need for symptomatic asthma therapy or SGCs. His total IgE level decreased to 860 IU/mL, and blood eosinophil count decreased to 290 cells/µL. However, 2 months after the initiation of therapy, conjunctivitis developed, which was temporarily alleviated by the use of GCS eye drops (Supplementary Figure S2A). In addition, rashes on the face and neck persisted throughout the treatment period (Supplementary Figures S2B,C).

Following a 28-month course of dupilumab, the medical commission added 300 mg of omalizumab, administered subcutaneously on a monthly basis. The dosage of the medication was selected independently of the total IgE level and the patient's body weight, based on the limited data of positive results in other studies (25–29) (Supplementary Table S1).

The combination therapy of dupilumab and omalizumab resulted in complete resolution of the conjunctivitis and regression of the facial rashes (Supplementary Figure 2D, scale for clinical case 2). Finally, 104 weeks after the initiation of combination therapy with dupilumab and omalizumab, the patient’s total IgE level decreased to 443 IU/mL.

## Clinical case 3

Patient G (female, 39 years old) presented to an allergist with the prevailing complaints of pruritic rashes on the trunk, extremities, face, and neck; recurrent attacks of dyspnea; and rhinoconjunctivitis upon contact with allergens.

The patient has been diagnosed with atopic dermatitis since childhood, managed by both an allergist and a dermatologist, which remitted from 20 to 25 years old and then relapsed after pregnancy. Emollients and topical GCS were ineffective.

The rhinoconjunctivitis has also been diagnosed since childhood and is triggered by environmental allergens [dust, dander, pollen, seasonal allergens (Apr/May), and snowmelt]. Birch slASIT (3 years) improved her symptoms related to pollen and decreased antihistamine use.

Asthma was diagnosed at the age of 6. For the past 5 years, the patient has been using a salmeterol/fluticasone inhaler (50/250 mcg) twice a day as background therapy, with the need for SABA up to 1–2 times a week.

A blood test indicated an elevated eosinophil count (up to 1,040 cells/mL) and total IgE level (up to 3,836 IU/mL). Spirometry revealed no abnormalities in ventilation. The concentration of nitric oxide in the exhaled air was 22 ppb.

Molecular allergocomponent diagnostics using the ALEX^2^ allergochip showed sensitization to epidermal (dog, cat, mouse, rat, rabbit, and horse), fungal (*Malassezia sympodialis* and *A. alternata*), pollen (tree, cereal, and weed pollen), and food (peanuts, apple, potato, hazelnut, egg protein, and yolk) allergens (Supplementary Table S1; Supplementary Figure S1.3).

Upon dupilumab initiation, comprehensive assessment of the AtD course (Supplementary Table S1), asthma control, and quality of life resulted in ACT, 23 points; ACQ5, 2 points; AQLQ, 149 points, respectively, which corresponds to partially controlled asthma with a lowered quality of life.

Dupilumab 300 mg subcutaneously every 2 weeks was initiated. Within 6 months, the patient showed improvement: the rashes gradually regressed, skin dryness and itching lessened, asthma attacks ceased, and no systemic GCS were required. Total IgE fell to 706 IU/mL and blood eosinophils to 760 cells/µL. Facial rashes persisted throughout treatment (Supplementary Figure S3A).

After 19 months of dupilumab treatment, the medical commission added 300 mg of omalizumab subcutaneously per month. The dose was selected according to the instructions for use, considering concomitant asthma and allergic rhinitis (30) (Supplementary Table S1).

Two months after the initiation of omalizumab, the combination therapy resulted in regression of the rashes in the facial area (Supplementary Figure S3B, scale for clinical case 3).

## Clinical case 4

Patient T (male, 24 years old) presented to an allergist with complaints of generalized rashes throughout the skin, debilitating itching that interfered with nighttime sleep, episodes of suffocation, and rhinoconjunctivitis upon contact with causally significant allergens.

The patient’s early-onset atopic dermatitis, managed by an allergist/dermatologist, follows a relapsing-remitting course without sustained remission. Exacerbations were treated with short-term SGCs, with consistent use of emollients and topical GCS.

Since childhood, the patient has been diagnosed with allergic rhinoconjunctivitis upon contact with household dust, animal hair, pollen, and seasonal allergens (in April–September). Facial and laryngeal angioedema manifests upon ingestion of buckwheat and nuts.

In 2019, methotrexate was prescribed (15 mg once a week) for severe, continuously recurring atopic dermatitis. Six months of therapy had a positive effect. However, the therapy was discontinued due to side effects (nausea and abdominal pain).

Dupilumab (600 mg SC loading dose, then 300 mg SC q2w) was initiated in May 2020 but discontinued in December 2021 due to recurrent, unresponsive acute marginal keratitis (treated by an ophthalmologist). Moreover, dupilumab caused pronounced facial and neck hyperemia and skin peeling, refractory to topical calcineurin inhibitors.

At the beginning of 2022, acute bronchitis resulted in wheezing and whistling in the patient’s chest and shortness of breath, and he was diagnosed with asthma by a pulmonologist. The current background therapy is beclomethasone/formoterol 100/6 mcg, with one inhalation two times a day and no need for SABA.

In April 2022, upadacitinib 15 mg/day was initiated, with a partial response. The dose increased to 30 mg/day in June 2022, leading to complete rash clearance. In December 2022, urgent hospitalization was required for Kaposi's herpetiform eczema complicated by pyoderma. The treatment included systemic/local antibiotics, GCS, antivirals, and supportive care, with positive results. Upadacitinib was stopped.

Following this, no monoclonal antibodies were prescribed; the disease deteriorated progressively, with generalized rashes and unbearable skin itching. The patient used topical GCS and antihistamines, with no effect.

A blood test indicated an elevated eosinophil count (up to 1,720 cells/µL) and total IgE level (8,895 IU/mL). Spirometry revealed no abnormalities in ventilation, and a test with a bronchodilator was negative. The level of nitric oxide in exhaled air reached 18 ppb.

Molecular allergocomponent diagnostics using the ALEX^2^ allergochip demonstrated sensitization to pollen (tree, cereal, and weed), epidermal (dog, cat, rabbit, mouse, and rat), fungal (*Malassezia*, baker's yeast, and *Aspergillus fumigatus*), and food (lentils, buckwheat, kiwi, apple, peach, celery, carrot, potato, nuts, sesame, egg, fish, and animal meat) allergens (Supplementary Table S1; Supplementary Figure S1.4).

A comprehensive assessment of the AtD course revealed a severe case of diffuse atopic dermatitis and significantly lowered quality of life (Supplementary Table S2); the patient’s asthma was controlled without compromising his quality of life: ACT, 20 points; ACQ5, 0.8 points; and AQLQ, 210 points.

In March 2023, targeted therapy with dupilumab was initiated at a starting dose of 600 mg subcutaneously once, followed by 300 mg once every 2 weeks.

One month after treatment initiation, rashes and itching decreased, improving his QoL. However, 2 weeks after dupilumab initiation, conjunctivitis developed (resistant to ophthalmologist-prescribed antihistamines/GCS), despite skin improvement. One month after dupilumab initiation, pronounced facial dermatitis emerged (Supplementary Figures S4A,B).

In April 2023, the medical commission added 300 mg of omalizumab subcutaneously per month. The dose of the medication was chosen regardless of the total IgE level and the patient's body weight, based on the limited data of positive results in other studies (25–29) (Supplementary Table S1).

The combination therapy, 1 month after the initiation of omalizumab, resolved the conjunctivitis completely and regressed the facial rashes (Supplementary Figure S4C, scale for clinical case 4).

Finally, 104 weeks after the initiation of combination therapy with dupilumab and omalizumab, the patient’s total IgE level decreased to 1,136 IU/mL.

## Discussion

This series of clinical cases describes AEs that occurred upon dupilumab treatment of atopic dermatitis, which have both been previously reported (conjunctivitis, 10) and not reported (6) in Phase III clinical trials. Despite the lack of data on the exact causes of these adverse events, sensitization to *Malassezia* was confirmed in all the clinical cases before starting dupilumab, corresponding to the previous data from Kozera et al. (21).

Blocking the Th2 pathway using biological therapies such as dupilumab is a successful therapeutic strategy in patients with AtD (31). This type of inflammatory pathway is involved in the homeostasis between the human microbiome and colonizing microorganisms. These include the fungal family *Malassezia* spp. (32, 33). The skin changes seen in AtD allow *Malassezia* spp. to colonize the skin more easily. Сolonization of *Malassezia* spp. on the skin induces the release of cytokines, such as tumor necrosis factor-alpha (TNF-α), IL-1β, and IL-18 (without IL-12). This leads to antigen presentation to Th2 lymphocytes, with the release of IL-13 (34). This cytokine maintains homeostasis between the organism and colonization by *Malassezia* spp. Blocking the Th2 pathway with drugs such as dupilumab induces an immune shift with increased activation of the Th17/Th22-mediated pathway. It has been shown in mouse models that overexpression of Th17/Th22 mediates the inflammatory hypersensitivity reaction to *Malassezia furfur* fungal proteins (35).

Considering the emerging data on the successful and safe use of several monoclonal antibody combinations in patients with asthma (24, 36), the simultaneous use of anti-IL-4 and anti-IgE therapy in patients with atopic dermatitis with pronounced adverse events has also been suggested to be effective and safe. In this study, we did not observe any significant increases in peripheral blood eosinophil levels. Eosinophil levels were measured 1 month after the initiation of therapy, and then every 3 months. Due to the increased levels of IgE specific to *Malassezia* and total IgE, omalizumab was chosen as an additional drug for biological therapy in these case studies.

The decrease in total immunoglobulin E levels during long-term treatment with dupilumab was predictable (37) and allowed for the prescription of omalizumab to one of the four patients at a dose calculated in accordance with the tabulated values from the package insert. For the remaining three patients, the dose was chosen independently of the patient's weight and total IgE level in the serum and the patient's weight, given the positive results after administering omalizumab for the treatment of atopic dermatitis and conjunctivitis in other studies (25–29).

In all the patients, the combination therapy regimen eliminated dupilumab-associated AEs in 1 or 2 months and prevented the occurrence of other adverse events. The monitoring of these patients and the combination therapy regimens continue to this day, with no adverse events reported. Additionally, there has been progressive improvement in their condition, as evidenced by validated questionnaires.

In the follow-up period, all the patients continued to receive a combination therapy with omalizumab and dupilumab. The dupilumab treatment regimen was maintained in all cases without extending the intervals, and no adverse events were observed with the concomitant use of the two biological immunomodulatory drugs thereafter.

This case series has several important limitations that should be acknowledged. First, the analysis does not include a formal assessment of treatment costs. Omalizumab is a biological therapy with a substantial financial burden, and its high cost may limit accessibility and long-term adherence in real-world settings. Economic factors, such as insurance coverage, out-of-pocket expenses, and healthcare system reimbursement policies, can significantly influence treatment decisions and outcomes, yet these were not evaluated in this study.

Second, while omalizumab is generally well-tolerated, it carries potential side effects that warrant consideration. The most commonly reported adverse events include injection-site reactions (e.g., pain, erythema, and swelling), headache, and fatigue. More rarely, omalizumab has been associated with anaphylaxis (occurring in <0.2 % of patients) and malignancy, although a causal relationship remains uncertain. Our case series did not systematically collect or report adverse events, which limits our ability to characterize the safety profile in this cohort. Future studies with structured safety monitoring would help clarify the risk–benefit balance in similar patient populations.

Third, the retrospective, single-center design and small sample size restrict the generalizability of our findings. Larger, prospective, multicenter studies are needed to confirm these observations and better quantify both the economic and safety implications of omalizumab use in this context.

The simultaneous use of omalizumab and dupilumab targets different molecular pathways involved in allergic and inflammatory diseases, potentially enhancing therapeutic efficacy (38).

This dual approach may be particularly beneficial in conditions where both IgE-mediated and Th2-cytokine-driven inflammation are prominent, such as asthma, bullous pemphigoid, or severe atopic dermatitis, the latter of which was demonstrated by Silva et al. (39). For example, in a case report of refractory bullous pemphigoid, the combination of omalizumab and dupilumab led to complete remission, likely due to the inhibition of IgE-autoantibodies and the suppression of IL-4/IL-13-mediated inflammation (40).

In summary, the simultaneous use of omalizumab and dupilumab leverages their distinct mechanisms to target multiple pathways in allergic and inflammatory diseases. While the evidence is still emerging, this combination shows promise in refractory cases but requires careful clinical management and monitoring.

## Conclusion

The severity of adverse events associated with dupilumab treatment may result in the discontinuation of the therapy and a loss of control over the atopic dermatitis. It is therefore important to identify the causes of the adverse events and implement further strategies to mitigate them. The expansion of indications for monoclonal antibody therapy and a combination of these drugs can be employed to control disease that is resistant to a single monoclonal antibody and to relieve the side effects of monotherapy. However, this combined biological therapy needs further monitoring of efficacy and safety.

## Data Availability

The raw data supporting the conclusions of this article will be made available by the authors, without undue reservation.
