# Supplementary material for: Case Series: Combination of dupilumab and omalizumab as a way to reduce dupilumab-associated adverse events in severe atopic dermatitis
Source: Front Allergy. 2026 Jan 16;6:1696897. doi: 10.3389/falgy.2025.1696897 (PMC12855484; doi:10.3389/falgy.2025.1696897)
Supplement: Supplementary file 1 [file Datasheet1.pdf]

Supplementary materials

Table 1.Patient background.

	Patient 1	Patient 2	Patient 3	Patient 4
Gender (Male/Female)	M	M	F	M
Age, years	40	23	39	24
Th-2 comorbidity	Asthma, ARC	Asthma, ARC	Asthma, ARC	Asthma, ARC
Blood eosinophil count on Dupilumab initiation (cells per microliter)	950	30	1040	1720
Blood eosinophil count on Omalizumab initiation (cells per microliter)	1940	10	710	890
Total IgE on Dupilumab initiation (ME/mL)	2786	>2000	3836	8895
Total IgE on Omalizumab initiation (kU/L)*	2694	860	98	2029
Patient's weight on Omalizumab initiation (kg)	86	77	58	67
IgE Malassezia sympodialis (kUA/L)				
Mala s 5	≤0.10	8.44	≤0.10	≤0.10
Mala s 6	2.61	2.45	3.87	≤0.10
Mala s 11	0.56	28.08	≤0.10	42.04

ARC, allergic rhinoconjunctivitis. *IgE ALEX2® Allergy Test.

Picture 1.1. Spectrum of sensitization for Clinical case 1. Pareto chart for patient 1.

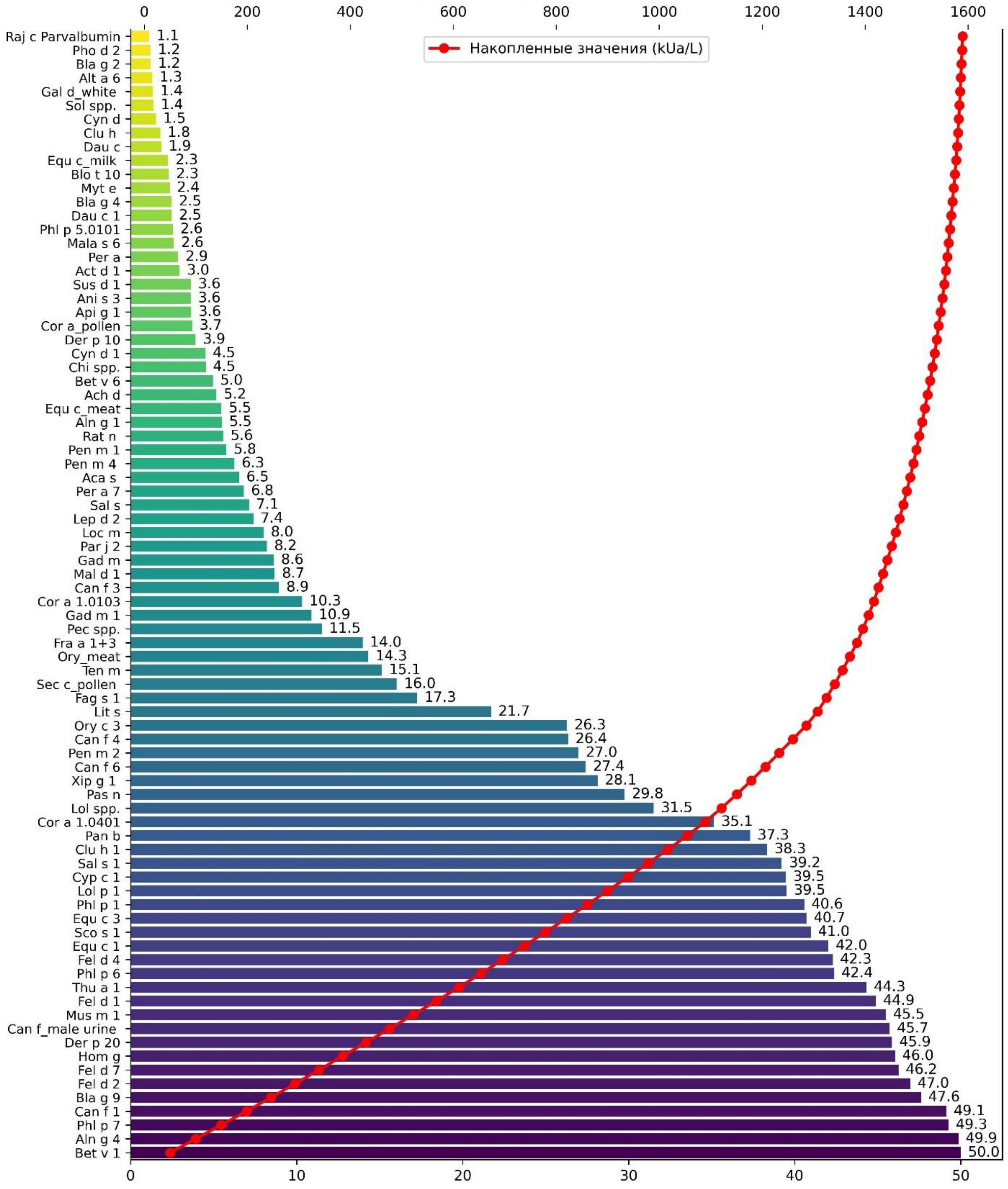

Picture 1.2. Spectrum of sensitization for Clinical case 2. Pareto chart for patient 2.

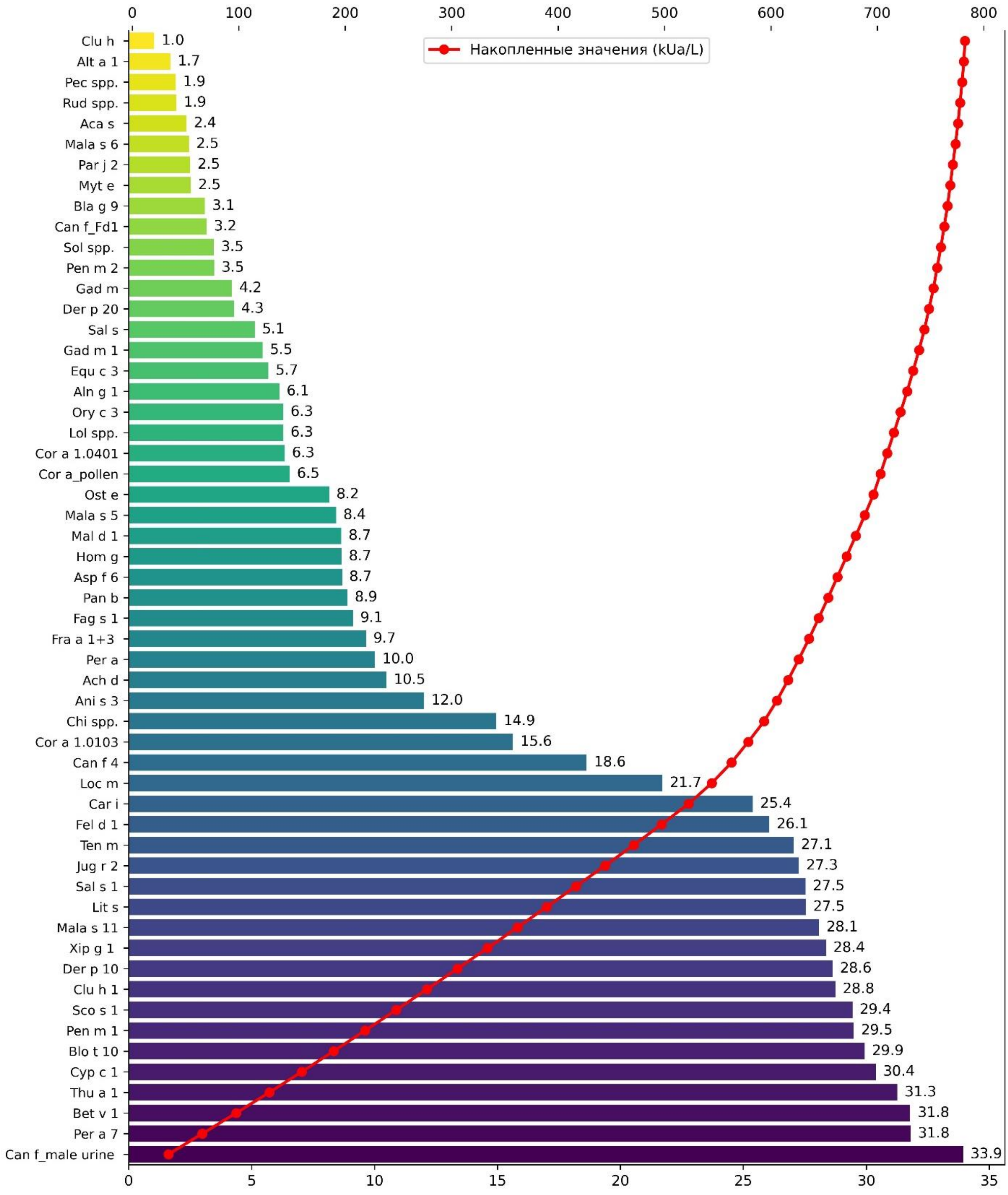

Picture 1.3. Spectrum of sensitization for Clinical case 3. Pareto chart for patient 3.

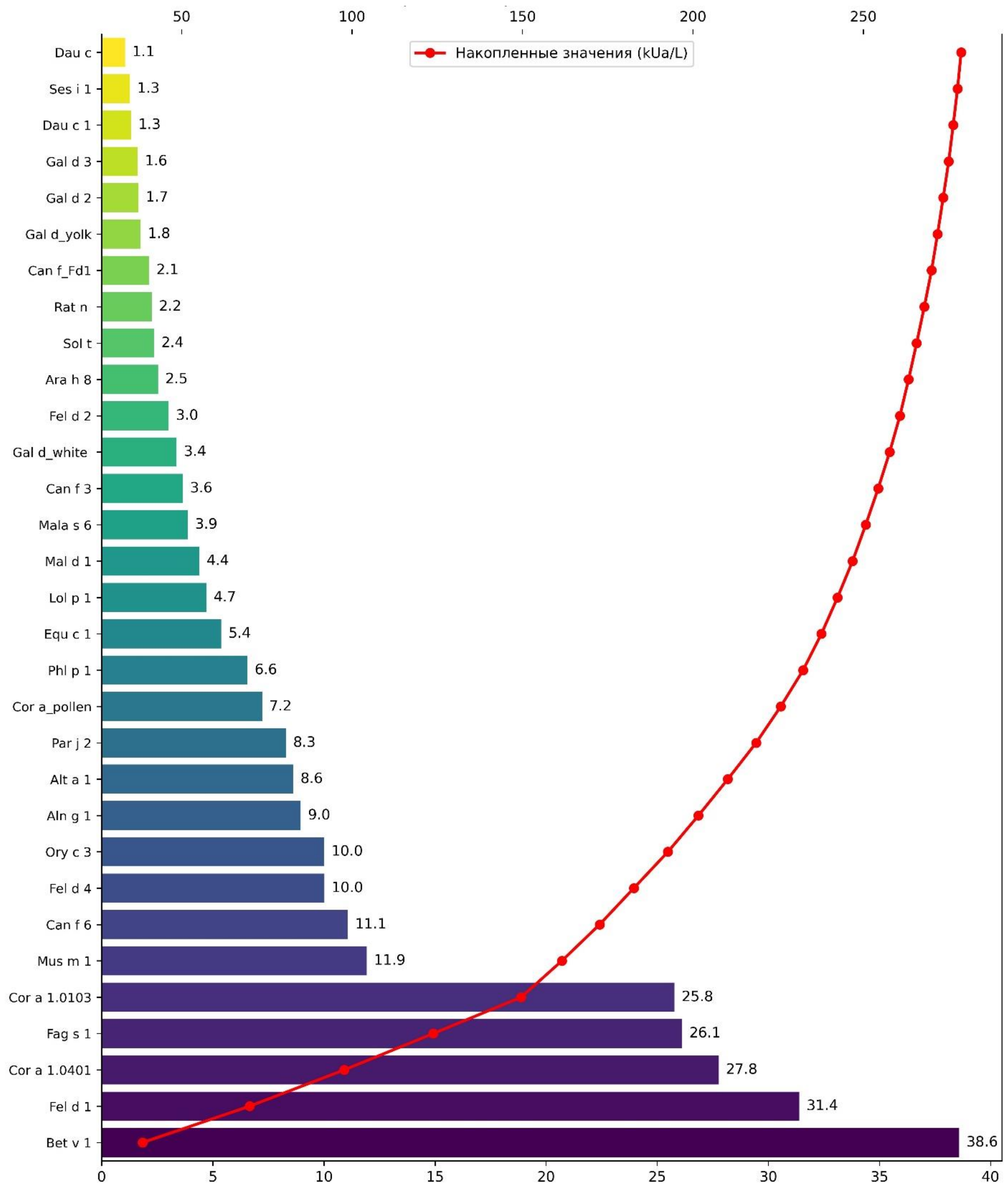

Picture 1.3. Spectrum of sensitization for Clinical case 4. Pareto chart for patient 4

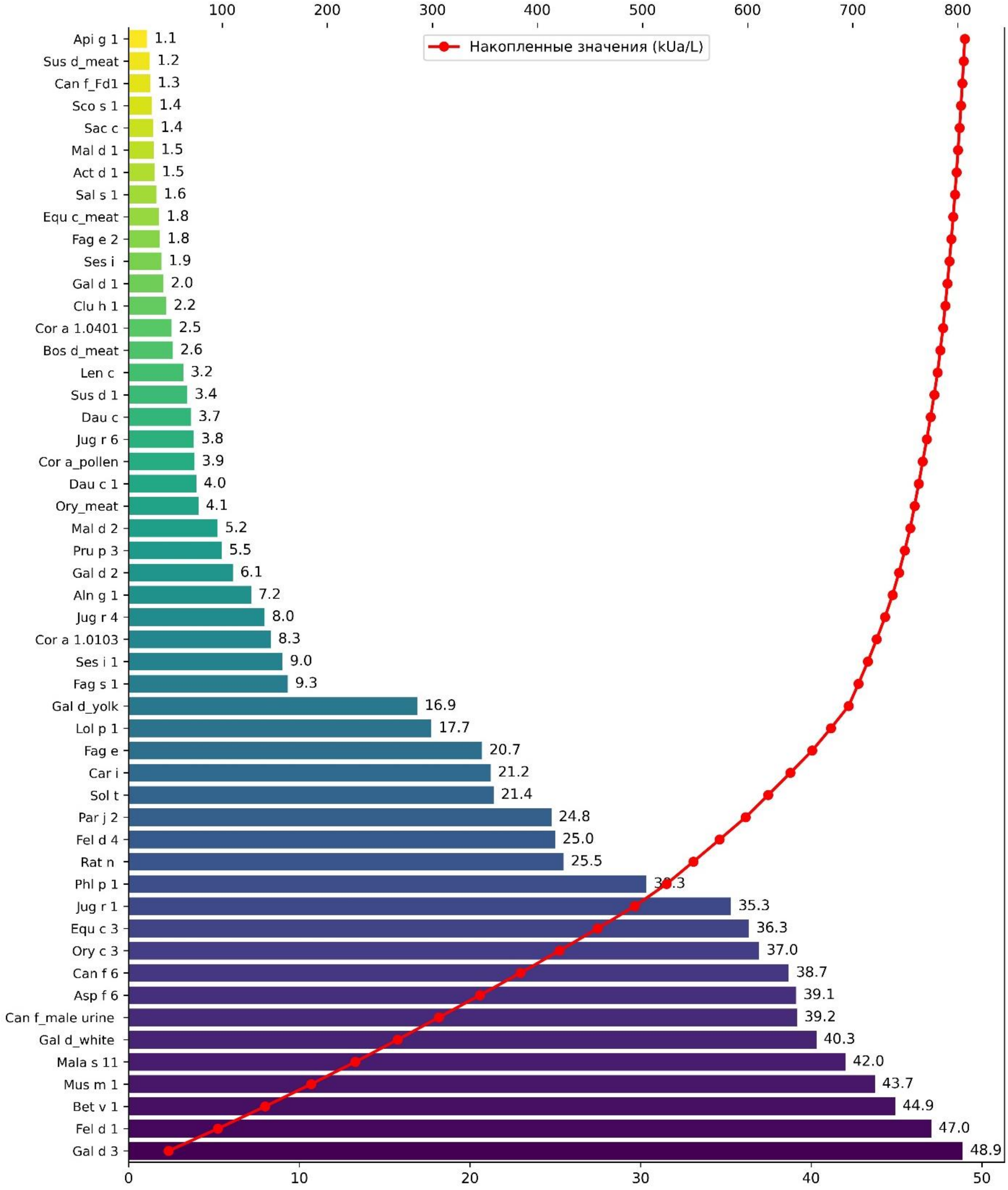

Table 2. Results of validated questionnaires

	Patient 1	Patient 2	Patient 3	Patient 4
Date of Dupilumab initiation	March 2023	December 2020	June 2021	March 2023
SCORAD on Dupilumab initiation	82.8	83	68.2	85.6
EASI on Dupilumab initiation	45.3	37.2	33.6	42.8
BSA on Dupilumab initiation	69	100	42	98
POEM on Dupilumab initiation	16	27	14	23
DQLI on Dupilumab initiation	12	24	10	20
Dupilumab-induced AEs, terms:<br>Conjunctivitis<br><br>Face and neck dermatitis	3 month after initiation<br><br>None	2 months after initiation<br><br>Throughout the treatment	None<br><br>Throughout the treatment	2 weeks after initiation<br><br>1 month after initiation
Time of Omalizumab adding/Months from Dupilumab initiation	September 2023/6 months	April 2023/28 months	January 2023/19 months	April 2023/ 1 month
SCORAD on Omalizumab initiation	45	37.4	34.2	79

EASI on Omalizumab initiation	10.8	10.6	4.8	37.6
BSA on Omalizumab initiation	20	27	21	91
POEM on Omalizumab initiation	11	12	4	24
DQLI on Omalizumab initiation	8	10	2	21
Dose and multiplicity of Omalizumab treatment	300 mg per month	300 mg per month	300 mg per month	300 mg per month

AE, adverse events, SCORAD, Scoring of Atopic Dermatitis, EASI, Eczema Area and Severity Index, BSA, Body Surface Area, POEM, Patient-Oriented Eczema Measure, DQLI, Dermatological Quality of Life Index

*IgE ALEX2® Allergy Test.

Scale for clinical case 1.

- Patient I. (1) 40 y.o

Comorbities: BA, ARC
Sensitization: HS, ES, FS, PS, FA

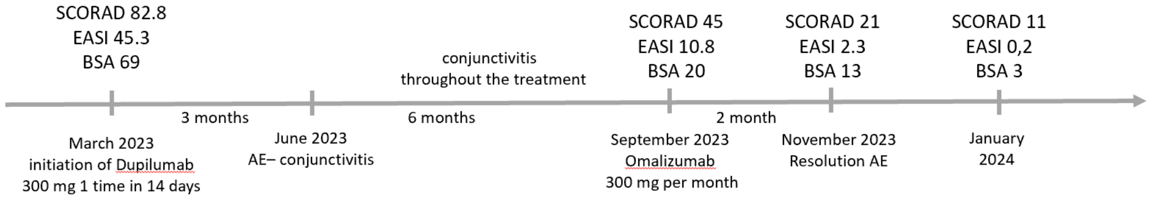

Spectrum of sensitization: HS- household sensitization, ES -epidermal sensitization, FS - fungal sensitization, PS -pollen sensitization, FA -food allergy. BA -bronchial asthma, ARC -allergic rhinoconjunctivitis. AE-adverse event.

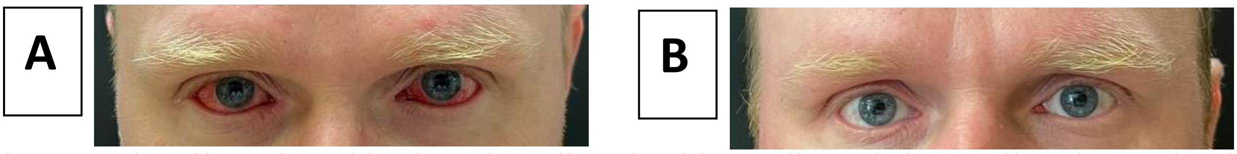

Figure 1. The effect of combination of Dupilumab with Omalizumab for Dupilumab-associated adverse events treatment in clinical case 1. A – conjunctivitis upon 4 month of Dupilumab treatment; B - regression of conjunctivitis 2 month upon start of combination therapy with Dupilumab and Omalizumab.

Scale for clinical case 2.

- Patient T. (2) 23 y.o

Comorbities: BA, ARC
Sensitization: HS, ES, FS, PS, FA

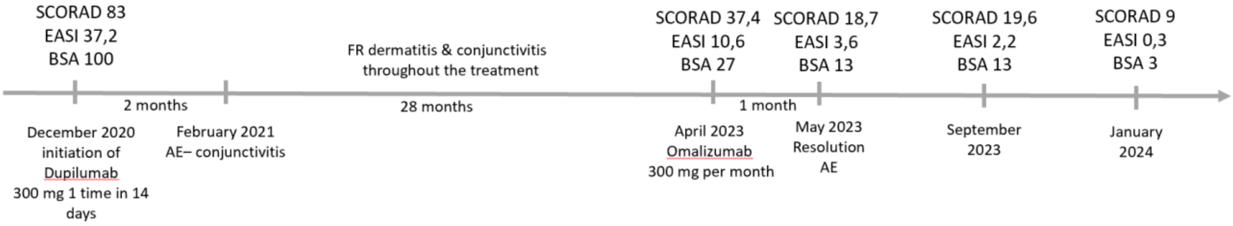

Spectrum of sensitization: HS- household sensitization, ES -epidermal sensitization, FS - fungal sensitization, PS -pollen sensitization, FA -food allergy. BA -bronchial asthma, ARC -allergic rhinoconjunctivitis. AE-adverse event.

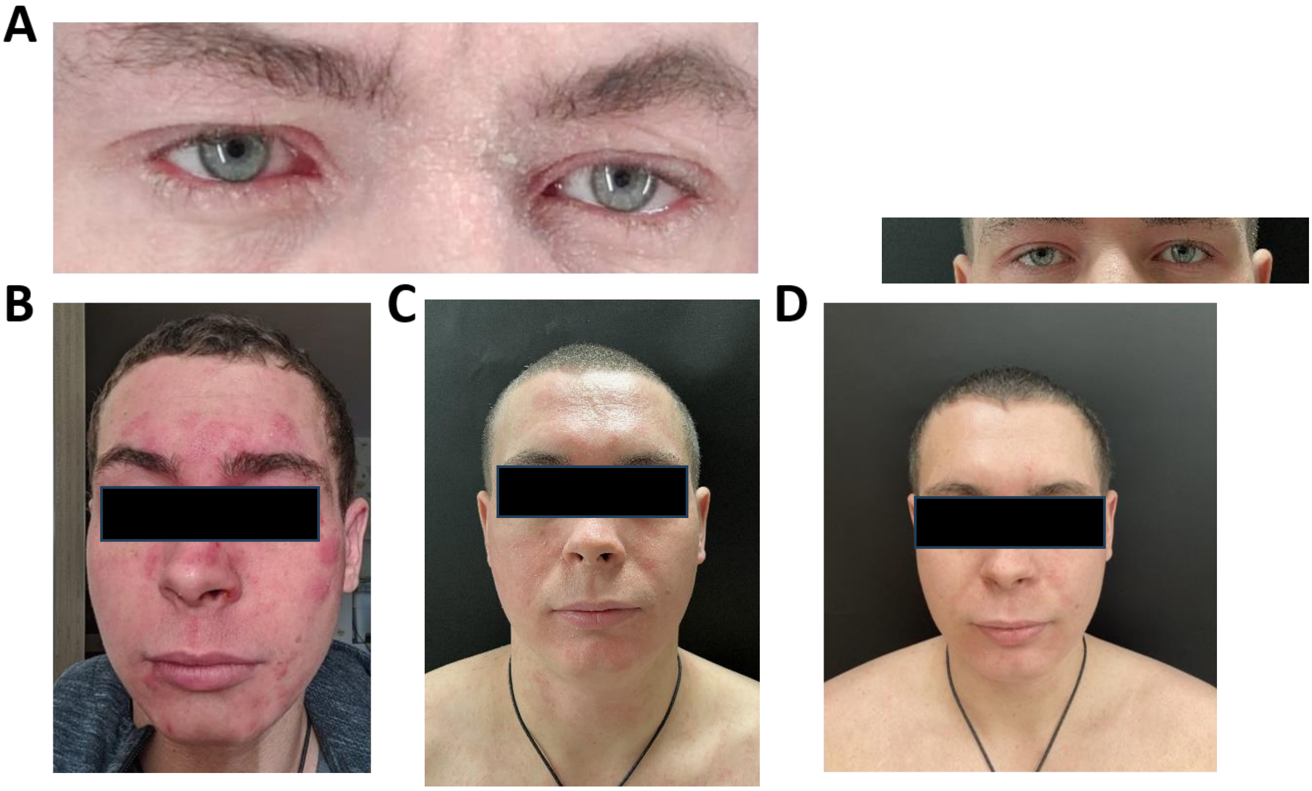

Figure 2. The effect of combination of Dupilumab with Omalizumab for Dupilumab-associated adverse events treatment in clinical case 2. A – conjunctivitis upon 2 month of Dupilumab treatment; B,C – face and neck rashes upon Dupilumab treatment; D – regression of conjunctivitis and rashes 1 month upon start of combination therapy with Dupilumab and Omalizumab.

Scale for clinical case 3.

- Patient G. (3) 39 y.o

Comorbities: BA, ARC
Sensitization: HS, ES, FS, PS, FA

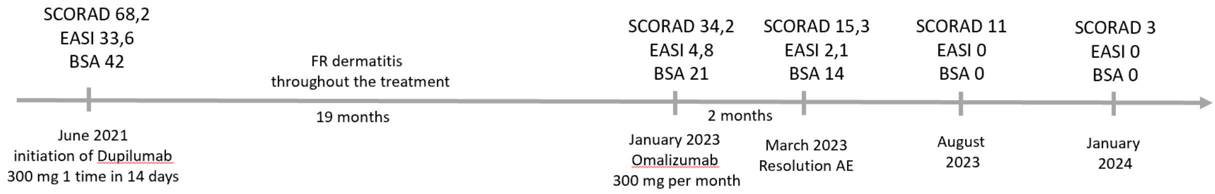

Spectrum of sensitization: HS- household sensitization, ES -epidermal sensitization, FS - fungal sensitization, PS -pollen sensitization, FA -food allergy. BA -bronchial asthma, ARC -allergic rhinoconjunctivitis. AE-adverse event.

A 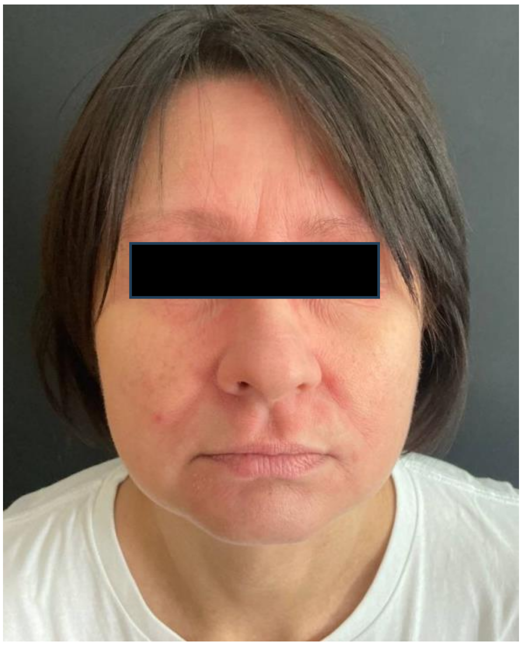 B 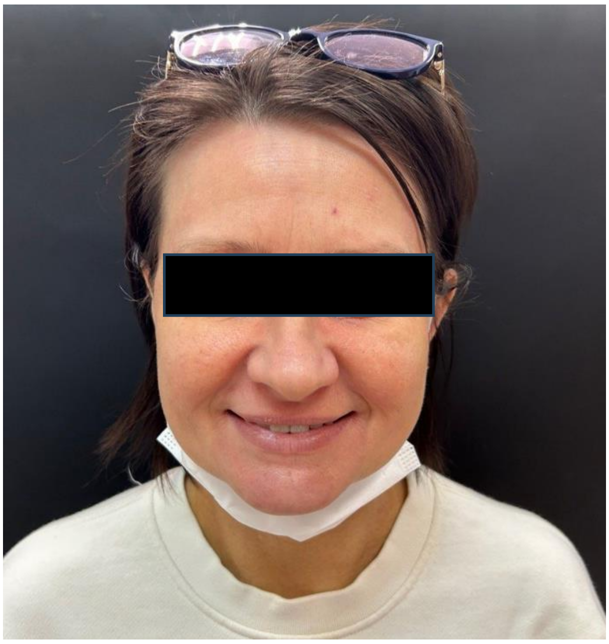

Figure 3. The effect of combination of Dupilumab with Omalizumab for Dupilumab-associated adverse events treatment in clinical case 3. A- Facial rashes upon treatment with Dupilumab in clinical case 3. B – regression of rashes 2 month upon start of combination therapy with Dupilumab and Omalizumab.

Scale for clinical case 4.

- Patient T. (4) 24 y.o

Comorbities: BA, ARC
Sensitization: HS, ES, FS, PS, FA

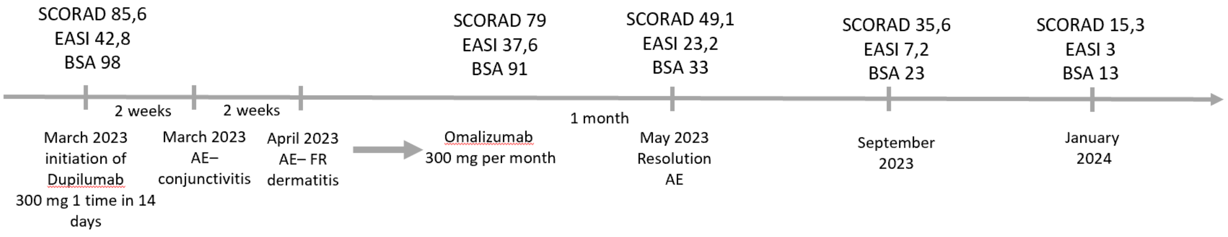

Spectrum of sensitization: HS- household sensitization, ES -epidermal sensitization, FS - fungal sensitization, PS -pollen sensitization, FA -food allergy. BA -bronchial asthma, ARC -allergic rhinoconjunctivitis. AE-adverse event.

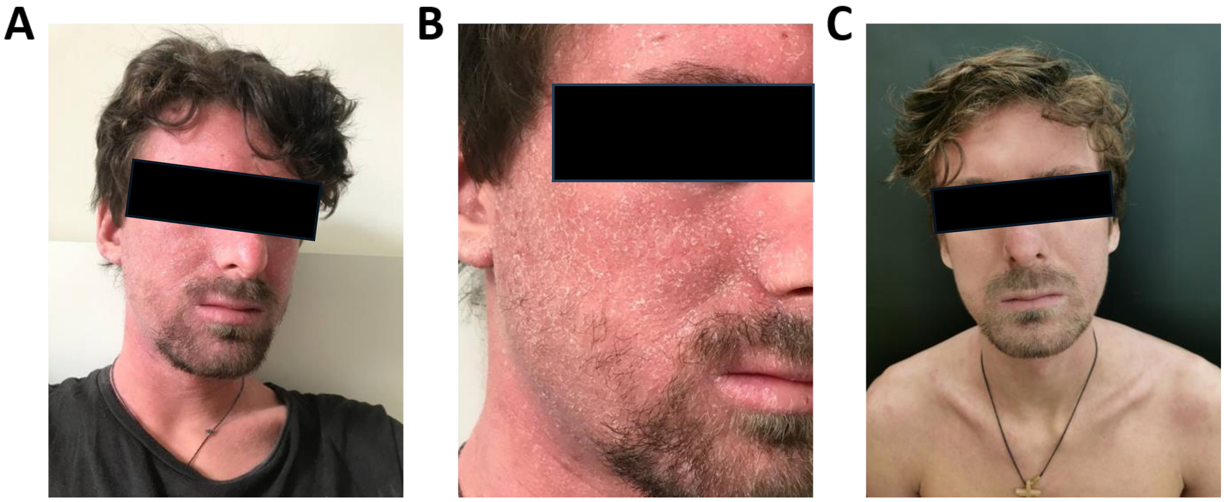

Figure 4 The effect of combination of Dupilumab with Omalizumab for Dupilumab-associated adverse events treatment in clinical case 4. A, B – facial dermatitis upon Dupilumab treatment; C – regression of dermatitis 1 month upon start of combination therapy with Dupilumab and Omalizumab.
